# *In Vitro* Induction of Erythrocyte Phosphatidylserine Translocation by the Natural Naphthoquinone Shikonin

**DOI:** 10.3390/toxins6051559

**Published:** 2014-05-13

**Authors:** Adrian Lupescu, Rosi Bissinger, Kashif Jilani, Florian Lang

**Affiliations:** Department of Physiology, University of Tuebingen, Gmelinstreet 5, Tuebingen 72076, Germany; E-Mails: lupescuadrian@gmx.de (A.L.); ro.bissinger@gmx.de (R.B.); kashif_cbc@yahoo.com (K.J.)

**Keywords:** phosphatidylserine, shikonin, calcium, ceramide, cell volume, eryptosis

## Abstract

Shikonin, the most important component of *Lithospermum erythrorhizon*, has previously been shown to exert antioxidant, anti-inflammatory, antithrombotic, antiviral, antimicrobial and anticancer effects. The anticancer effect has been attributed to the stimulation of suicidal cell death or apoptosis. Similar to the apoptosis of nucleated cells, erythrocytes may experience eryptosis, the suicidal erythrocyte death characterized by cell shrinkage and by phosphatidylserine translocation to the erythrocyte surface. Triggers of eryptosis include the increase of cytosolic Ca^2+^-activity ([Ca^2+^]_i_) and ceramide formation. The present study explored whether Shikonin stimulates eryptosis. To this end, Fluo 3 fluorescence was measured to quantify [Ca^2+^]_i_, forward scatter to estimate cell volume, annexin V binding to identify phosphatidylserine-exposing erythrocytes, hemoglobin release to determine hemolysis and antibodies to quantify ceramide abundance. As a result, a 48 h exposure of human erythrocytes to Shikonin (1 µM) significantly increased [Ca^2+^]_i_, increased ceramide abundance, decreased forward scatter and increased annexin V binding. The effect of Shikonin (1 µM) on annexin V binding was significantly blunted, but not abolished by the removal of extracellular Ca^2+^. In conclusion, Shikonin stimulates suicidal erythrocyte death or eryptosis, an effect at least partially due to the stimulation of Ca^2+^ entry and ceramide formation.

## 1. Introduction

Shikonin, a naphthoquinone, is the most important component of *Lithospermum erythrorhizon*, a traditional Chinese herbal medicine [1]. Shikonin has antioxidant [1], anti-inflammatory [1,2,3], antithrombotic [1,2], antiviral [2,4], antimicrobial [1,2], as well as anticancer [2,5,6,7,8] potency and fosters wound healing [1,2,5]. The anticancer effect of Shikonin has been attributed at least in part to the stimulation of suicidal cell death or apoptosis [9,10,11,12,13,14,15,16,17]. Mechanisms involved in Shikonin-induced apoptosis include reactive oxidant species [18,19,20,21,22,23], altered gene expression [15], protein phosphorylation [18] and caspase activation [24].

Similar to the apoptosis of nucleated cells, erythrocytes may undergo eryptosis, a suicidal erythrocyte death characterized by phosphatidylserine translocation and cell shrinkage [25]. Eryptosis is stimulated by increase of cytosolic Ca^2+^ concentration ([Ca^2+^]_i_), e.g., due to stimulation of Ca^2+^ entry [25]. Increased [Ca^2+^]_i_ leads to the activation of Ca^2+^-sensitive K^+^ channels with subsequent cell shrinkage due to K^+^ exit, hyperpolarization, Cl^−^ exit and thus to cellular loss of KCl and osmotically obliged water [26]. Increased [Ca^2+^]_i_ is further followed by phospholipid scrambling of the cell membrane with phosphatidylserine exposure at the erythrocyte surface [25]. Eryptosis may further be triggered by ceramide formation [25], caspase activation [27,28,29,30,31] and deranged activities of AMP activated kinase (AMPK) [32], casein kinase 1α [33,34], cGMP-dependent protein kinase [35], Janus-activated kinase (JAK3) [36], protein kinase C [37], p38 kinase [38], PAK2 kinase [39], and/or sorafenib- [40] and sunitinib- [41] sensitive kinases.

Eryptosis is triggered by a myriad of xenobiotics [25,41,42,43,44,45,46,47,48,49,50,51,52,53,54,55,56,57,58,59,60,61,62,63,64,65,66,67,68,69,70,71], and excessive eryptosis is observed in a wide variety of clinical conditions, such as diabetes, renal insufficiency, hemolytic uremic syndrome, sepsis, malaria, sickle cell disease, Wilson’s disease, iron deficiency, malignancy, phosphate depletion and metabolic syndrome [25].

The present study explored, whether and, if so, how Shikonin stimulates eryptosis. To this end, [Ca^2+^]_i_, cell volume and phosphatidylserine exposure were determined in the absence and presence of Shikonin.

## 2. Results and Discussion

**Figure 1:**
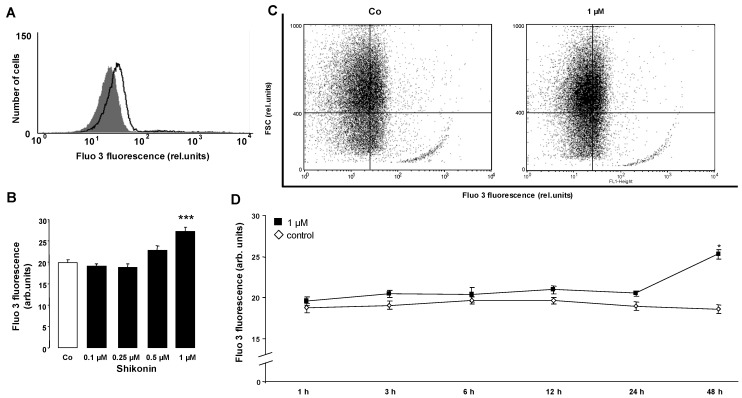
The effect of Shikonin on erythrocyte cytosolic Ca^2+^ concentration. (**A**) Original histogram of the Fluo 3 fluorescence in erythrocytes following exposure for 48 h to Ringer solution without (grey area) and with (black line) the presence of 1 µM Shikonin; (**B**) Arithmetic means ± SEM (n = 8) of the Fluo 3 fluorescence (arbitrary units) in erythrocytes exposed for 48 h to Ringer solution without (white bar) or with (black bars) Shikonin (0.1–1 µM). *** (*p* < 0.001) indicates significant difference from the absence of Shikonin (ANOVA); (**C**) Original dot blots of the Fluo 3 fluorescence as a function of forward scatter following exposure for 48 h to Ringer solution without and with the presence of 1 µM Shikonin; (**D**) Arithmetic means ± SEM (n = 4) of the Fluo 3 fluorescence (arbitrary units) in erythrocytes exposed for 1–48 h to Ringer solution without (white squares) or with 1 µM Shikonin (black squares). * (*p* < 0.05) indicates significant difference from the absence of Shikonin (ANOVA).

The present study explored whether the naphthoquinone Shikonin triggers eryptosis, the suicidal erythrocyte death characterized by cell shrinkage and phosphatidylserine translocation. As both hallmarks of eryptosis are stimulated by the increase of cytosolic Ca^2+^ activity ([Ca^2+^]_i_), the effect of Shikonin on [Ca^2+^]_i_ was tested in a first series of experiments. To this end, human erythrocytes were loaded with Fluo3-AM and the Fluo 3 fluorescence determined by flow cytometry. Prior to the determination of Fluo 3 fluorescence, the erythrocytes were incubated for 48 h in Ringer solution without or with Shikonin (0.1–1 µM). As illustrated in Figure 1, the exposure to Shikonin was followed by an increase of Fluo 3 fluorescence, an effect reaching statistical significance at a 1-µM Shikonin concentration. Accordingly, Shikonin increased cytosolic Ca^2+^ concentration. 

**Figure 2:**
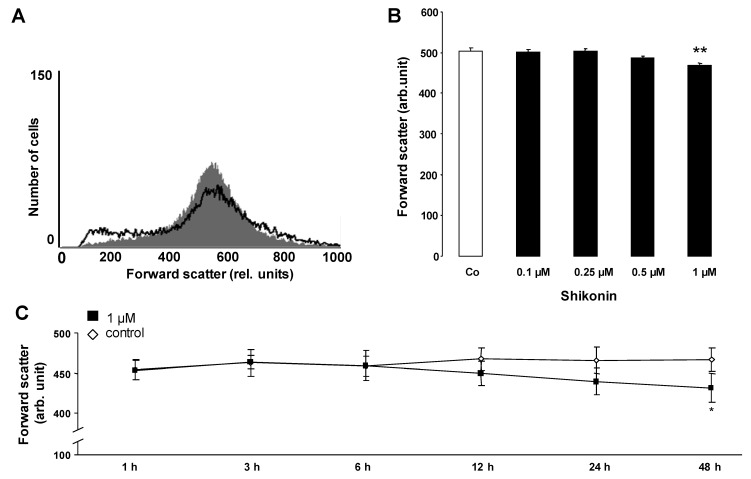
The effect of Shikonin on erythrocyte forward scatter. (**A**) Original histogram of the forward scatter of erythrocytes following exposure for 48 h to Ringer solution without (grey area) and with (black line) the presence of 1 µM Shikonin; (**B**) Arithmetic means ± SEM (n = 8) of the normalized erythrocyte forward scatter (FSC) following incubation for 48 h to Ringer solution without (white bar) or with (black bars) Shikonin (0.1–1 µM). ** (*p* < 0.01) indicates significant difference from the absence of Shikonin (ANOVA); (**C**) Arithmetic means ± SEM (n = 4) of the forward scatter (arbitrary units) in erythrocytes exposed for 1–48 h to Ringer solution without (white squares) or with 1 µM Shikonin (black squares). * (*p* < 0.05) indicates significant difference from the absence of Shikonin (ANOVA).

An increase of [Ca^2+^]_i_ may trigger cell shrinkage due to the activation of Ca^2+^-sensitive K^+^ channels and the subsequent exit of KCl and osmotically obliged water. Thus, cell volume was estimated from forward scatter in flow cytometry. As illustrated in Figure 2, a 48 h exposure to Shikonin was followed by a decrease of forward scatter, an effect reaching statistical significance at 1 µM Shikonin concentration. Accordingly, Shikonin treatment was followed by erythrocyte shrinkage. 

**Figure 3:**
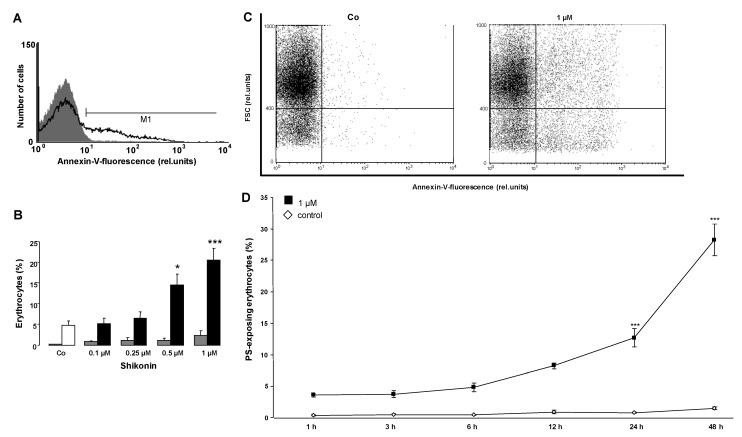
The effect of Shikonin on phosphatidylserine exposure*.* (**A**) Original histogram of the annexin V binding of erythrocytes following exposure for 48 h to Ringer solution without (grey area) and with (black line) the presence of 1 µM Shikonin; (**B**) Arithmetic means ± SEM of the erythrocyte annexin V binding (n = 8) following incubation for 48 h to Ringer solution without (white bar) or with (black bars) the presence of Shikonin (0.1–1 µM). For comparison, the arithmetic means ± SEM of hemolysis (n = 8) following incubation for 48 h to Ringer solution without or with the presence of Shikonin (grey bars) is shown. * (*p* < 0.05), *** (*p* < 0.001) indicate significant difference from the absence of Shikonin (ANOVA); (**C**) Original dot blots of the annexin V binding as a function of forward scatter following exposure for 48 h to Ringer solution without and with the presence of 1 µM Shikonin; (**D**) Arithmetic means ± SEM (n = 4) of the annexin V binding erythrocytes (arbitrary units) following exposure for 1–48 h to Ringer solution without (white squares) or with 1 µM Shikonin (black squares). *** (*p* < 0.001) indicates significant difference from the absence of Shikonin (ANOVA).

An increased [Ca^2+^]_i_ may further trigger cell membrane phospholipid scrambling with phosphatidylserine translocation to the erythrocyte surface. Phosphatidylserine exposing erythrocytes were identified utilizing annexin V binding as determined in flow cytometry. As illustrated in Figure 3, a 48 h exposure to Shikonin was followed by an increase of the percentage of annexin V binding erythrocytes, an effect reaching statistical significance at a 0.5 µM Shikonin concentration. Accordingly, Shikonin triggered erythrocyte phosphatidylserine translocation with the subsequent translocation of phosphatidylserine to the cell surface.

Further experiments were performed to quantify the effect of Shikonin on hemolysis. The percentage of hemolyzed erythrocytes was calculated from the hemoglobin concentration in the supernatant. As illustrated in Figure 3, the percentage of hemolyzed erythrocytes did not significantly increase following Shikonin exposure.

**Figure 4:**
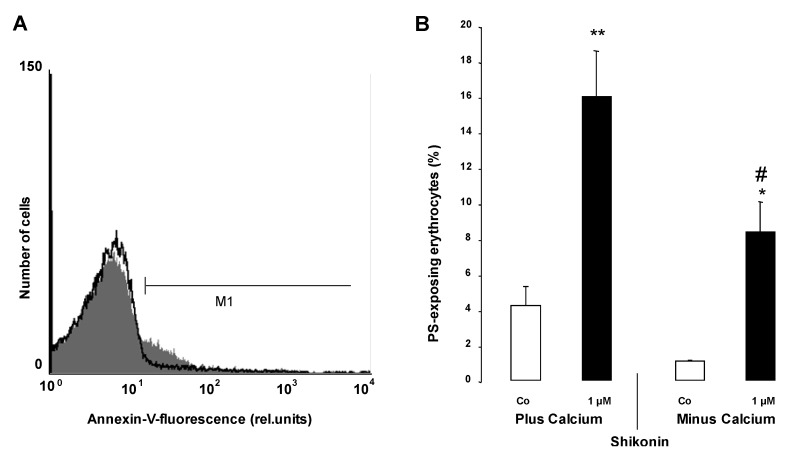
The effect of Ca^2+^ withdrawal on Shikonin-induced annexin V binding. (**A**) Original histogram of the annexin V binding of erythrocytes following exposure for 48 h to Ringer solution with (grey area) and without (black line) calcium in the presence of 1 µM Shikonin; (**B**) Arithmetic means ± SEM (n = 4) of the percentage of annexin V binding of erythrocytes after a 48 h treatment with Ringer solution without (white bar) or with (black bars) 1 µM Shikonin in the presence (left bars, plus calcium) and absence (right bars, minus calcium) of calcium. * (*p* < 0.05), ** (*p* < 0.01) indicate significant difference from the respective values in the absence of Shikonin; # (*p* < 0.05) indicates significant difference from the respective value in the presence of Ca^2+^ (ANOVA).

In order to test whether the Shikonin induced phosphatidylserine translocation required the entry of extracellular Ca^2+^, erythrocytes were treated for 48 h with 1 µM Shikonin in either the presence or nominal absence of extracellular Ca^2+^. As illustrated in Figure 4, the effect of Shikonin on annexin V binding was significantly blunted in the nominal absence of Ca^2+^. However, even in the nominal absence of Shikonin, the percentage of annexin V binding erythrocytes increased significantly. Thus, the effect of Shikonin was apparently not fully dependent on Ca^2+^ entry. 

**Figure 5:**
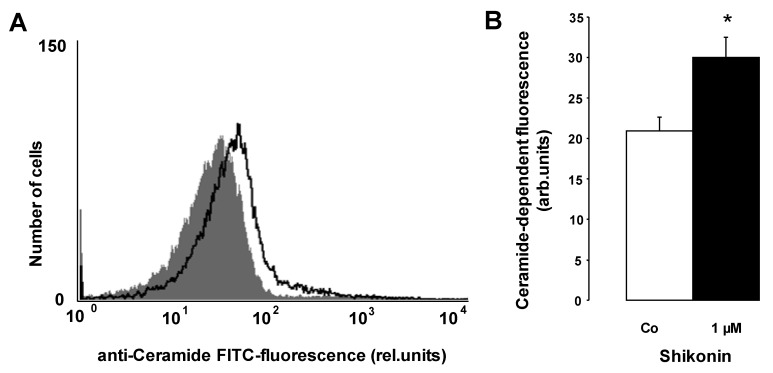
The effect of Shikonin on ceramide formation. (**A**) Original histogram of the ceramide surface abundance of erythrocytes following exposure for 48 h to Ringer solution without (grey shadow) and with (black line) the presence of 1 µM Shikonin. (**B**) Arithmetic means ± SEM (n = 4) of the ceramide abundance after a 48 h incubation in Ringer solution without (white bars) or with 1 µM Shikonin (black bars). * (p < 0.05) indicates significant difference from the absence of Shikonin (*t*-test).

In the search for an additional mechanism, further experiments were performed in order to test whether Shikonin increased the formation of ceramide. Ceramide abundance at the erythrocyte surface was determined utilizing an anti-ceramide antibody. As illustrated in Figure 5, the exposure of erythrocytes to 1 µM Shikonin significantly increased the ceramide abundance at the erythrocyte surface.

**Figure 6:**
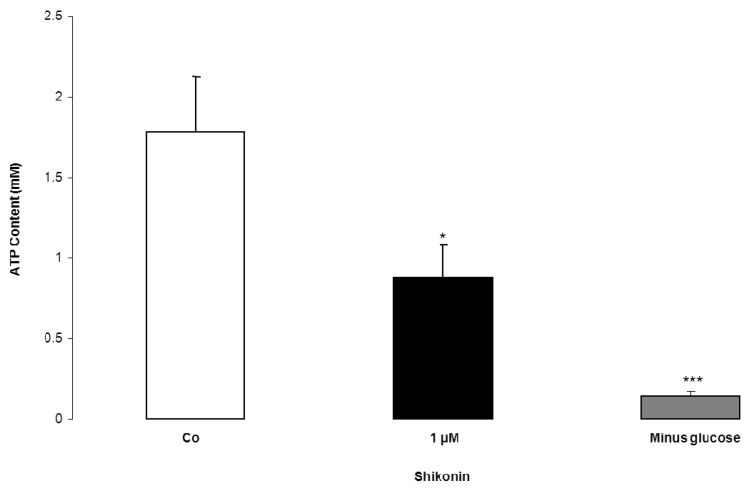
The effect of Shikonin on erythrocyte cytosolic ATP content. Arithmetic means ± SEM (n = 4) of the ATP concentration after a 48 h incubation in Ringer solution without (white bar) or with 1 µM Shikonin (black bar) or in glucose-depleted Ringer solution (grey bar, minus glucose) * (*p* < 0.05) and *** (*p* < 0.001) indicate significant difference from the control (absence of Shikonin and presence of glucose) (ANOVA).

Additional experiments tested the effect of Shikonin on erythrocytic ATP concentration. As illustrated in Figure 6, a 48 h incubation of erythrocytes with 1 µM Shikonin was followed by a significant decrease of erythrocytic ATP concentration. For a comparison, the effect of glucose removal on erythrocytic ATP concentration is shown in Figure 6. 

The present study reveals that Shikonin stimulates eryptosis, the suicidal death of erythrocytes [25]. The hallmark of eryptosis is phosphatidylserine translocation to the erythrocyte surface [25]. Eryptosis is further characterized by cell shrinkage [25]. Shikonin exposure of human erythrocytes leads to phosphatidylserine translocation and to cell shrinkage.

The Shikonin-induced cell shrinkage is presumably due to the increase of [Ca^2+^]_i_, which activates Ca^2+^-sensitive K^+^ channels [25], leading to K^+^ exit, cell membrane hyperpolarization, Cl^−^ exit and, thus, cellular loss of KCl with osmotically obliged water [26]. The cellular loss of KCl counteracts erythrocyte swelling, which may jeopardize the integrity of the erythrocyte membrane. Excessive erythrocyte swelling leads to hemolysis with the subsequent release of hemoglobin, which subsequently undergoes glomerular filtration and precipitation in the acidic tubular lumina [72].

Stimulation of phosphatidylserine translocation following Shikonin treatment is similarly a result of Ca^2+^ entry. Accordingly, the effect of Shikonin was significantly blunted in the nominal absence of Ca^2+^. Removal of Ca^2+^ decreased phosphatidylserine translocation even in the absence of Shikonin, indicating that spontaneous eryptosis following the exposure of erythrocytes to Ringer without Shikonin was similarly, in part, caused by cytosolic Ca^2+^. However, significant phosphatidylserine exposure was observed in the presence of Shikonin even in the nominal absence of Ca^2+^, indicating that Shikonin was effective by an additional mechanism. As a matter of fact, Shikonin triggered the formation of ceramide, which, in turn, fosters phosphatidylserine translocation [25]. Ceramide is formed by a sphingomyelinase, which is, in turn, activated by a platelet activating factor [25]. Ceramide fosters suicidal death similarly in nucleated cells [73]. It is effective by potentiating proapoptotic signaling [73]. Shikonin further decreases erythrocytic ATP concentration, another known trigger of eryptosis [25].

The molecular mechanism underlying phosphatidylserine translocation has not yet been identified [25]. Recently, Anoctamin 6 (Ano6; TMEM16F gene) has been suggested to mediate cell membrane scrambling [74]. The molecule may function as a Cl^−^ channel, a Ca^2+^-regulated nonselective Ca^2+^ permeable cation channel, a scramblase mediating phosphatidylserine translocation upon the increase of cytosolic Ca^2+^ and a regulator of cell membrane blebbing and microparticle shedding [74]. However, Ano6 is inhibited by tannic acid [75], which itself triggers phosphatidylserine translocation in erythrocytes [60]. Thus, the role of Ano6 in the regulation of phosphatidylserine translocation in the erythrocyte membrane remains elusive.

Stimulation of eryptosis may be favorable, as it allows the elimination of defective erythrocytes prior to hemolysis [25]. The phosphatidylserine exposure at the cell surface may be particularly important during the course of malaria, as infected phosphatidylserine exposing cells may be recognized and, thus, rapidly removed from circulating blood. [76]. Infected erythrocytes undergo eryptosis, as the intraerythrocytic parasite activates several ion channels, including the Ca^2+^-permeable erythrocyte cation channels [77,78]. Activation of the channels in the host cell membrane is required for the intraerythrocytic survival of the pathogen [77,78], as the channels provide the pathogen with nutrients, Na^+^ and Ca^2+^, and they mediate the disposal of intracellular waste products [78]. By the same token, the Ca^2+^ entry through the Ca^2+^-permeable cation channels triggers eryptosis [76], which is, in turn, followed by the rapid clearance of the infected erythrocytes from circulating blood [25]. Ca^2+^ entry and Ca^2+^-induced eryptosis thus lead to elimination, not only of the infected erythrocyte, but also of the pathogen [76].

Accordingly, genetic disorders predisposing to accelerated eryptosis, such as the sickle-cell trait, the beta-thalassemia trait, homozygous Hb-C and G6PD deficiency [25], lead to a relatively mild clinical course of malaria following infection with *Plasmodia* [79,80,81]. Moreover, several clinical conditions, such as iron deficiency [82], and eryptosis stimulating drugs, such as lead [83], chlorpromazine [84] or NO synthase inhibitors [85], have been shown to favorably influence the clinical course of malaria. It may be worth considering whether Shikonin similarly decreases parasitemia in malaria. At least in theory, Shikonin and further proeryptotic substances could be employed for the treatment of malaria. However, the applicability of Shikonin in the treatment of malaria has not been tested, and its clinical use may depend on further properties and side effects not studied here. 

Excessive stimulation of eryptosis may lead to anemia. Phosphatidylserine at the surface of eryptotic cells triggers the phagocytosis of the cells and, thus, leads to the rapid removal of the suicidal erythrocytes from circulating blood [25]. Anemia develops, if the accelerated clearance of erythrocytes during stimulated eryptosis outcasts the formation of new erythrocytes [25]. Phosphatidylserine exposing erythrocytes may further interfere with microcirculation [86,87,88,89,90,91]. The phosphatidylserine exposing cells adhere to endothelial CXCL16/SR-PSO [87] and may stimulate blood clotting and thrombosis [86,92,93]. 

Anemia and compromised microcirculation may, at least in theory, limit the use of Shikonin. The substance has been proposed to counteract oxidative stress [1], inflammation [1,2,3], thrombosis [1,2], infections [1,2] and cancer [2,5,6,7,8]. Moreover, Shikonin has been used to support wound healing [1,2,5]. However, the toxicity of Shikonin is still ill-defined [1]. The present observations point to a novel potentially toxic effect of Shikonin. The local application of the substance, such as in creams and ointments [1], is not expected to trigger eryptosis. Systemic application of the substance may, however, jeopardize erythrocyte survival and microcirculation, thus potentially limiting the use of the substance. This may particularly be true in patients suffering from diseases with enhanced eryptosis [25] or receiving other eryptosis-inducing xenobiotics [25,41,42,43,44,45,46,47,48,49,50,51,52,53,54,55,56,57,58,59,60,61,62,63,64,65,66,67,68,69,70,71]. After oral and intramuscular administration, Shikonin is rapidly absorbed and has a half-life in plasma of 8.8 h and a distribution volume of 8.91 L/kg [1]. Several approaches have improved the stability and water solubility of Shikonin, thus providing the basis for the systemic application of the substance [1]. Future studies will be required to define the impact of Shikonin on erythrocyte survival and microcirculation following systemic application of Shikonin.

## 3. Experimental Section

### 3.1. Erythrocytes, Solutions and Chemicals

Leukocyte-depleted erythrocytes were kindly provided by the blood bank of the University of Tübingen. The study is approved by the ethics committee of the University of Tübingen (184/2003V). Erythrocytes were incubated *in vitro* at a hematocrit of 0.4% in Ringer solution containing (in mM) 125 NaCl, 5 KCl, 1 MgSO_4_, 32 N-2-hydroxyethylpiperazine-N-2-ethanesulfonic acid (HEPES), 5 glucose, 1 CaCl_2_; pH 7.4 at 37°C for 48 h. Where indicated, erythrocytes were exposed to Shikonin (Enzo, Lörrach, Germany) at the indicated concentrations. In Ca^2+^-free Ringer solution, 1-mM CaCl_2_ was substituted by 1-mM glycol-bis(2-aminoethylether)-N,N,N',N'-tetraacetic acid (EGTA). 

### 3.2. Analysis of Annexin V Binding and Forward Scatter

After incubation under the respective experimental condition, a 50 µL cell suspension was washed in Ringer solution containing 5-mM CaCl_2_ and then stained with annexin V FITC (1:200 dilution; ImmunoTools, Friesoythe, Germany) in this solution at 37 °C for 20 min under protection from light. In the following, the forward scatter (FSC) of the cells was determined, and the annexin V fluorescence intensity was measured with an excitation wavelength of 488 nm and an emission wavelength of 530 nm on a FACS Calibur (BD, Heidelberg, Germany).

### 3.3. Measurement of Intracellular Ca^2+^

After incubation, erythrocytes were washed in Ringer solution and then loaded with Fluo-3/AM (Biotium, Hayward, USA) in Ringer solution containing 5-mM CaCl_2_ and 5-µM Fluo-3/AM. The cells were incubated at 37 °C for 30 min and washed twice in Ringer solution containing 5-mM CaCl_2_. The Fluo-3/AM-loaded erythrocytes were resuspended in 200 µL of Ringer. Then, Ca^2+^-dependent fluorescence intensity was measured with an excitation wavelength of 488 nm and an emission wavelength of 530 nm on a FACS Calibur.

### 3.4. Determination of Ceramide Formation

For the determination of ceramide, a monoclonal antibody-based assay was used. After incubation, cells were stained for 1 hour at 37 °C with 1 µg/mL of anti-ceramide antibody (clone MID 15B4, Alexis, Grünberg, Germany) in PBS containing 0.1% bovine serum albumin (BSA) at a dilution of 1:5. The samples were washed twice with PBS-BSA. Subsequently, the cells were stained for 30 minutes with polyclonal fluorescein isothiocyanate (FITC) conjugated goat anti-mouse IgG and IgM-specific antibody (Pharmingen, Hamburg, Germany) diluted 1:50 in PBS-BSA. Unbound secondary antibody was removed by repeated washing with PBS-BSA. The samples were then analyzed by flow cytometric analysis with an excitation wavelength of 488 nm and an emission wavelength of 530 nm. 

### 3.5. ATP Content

For the determination of the intracellular ATP concentration, erythrocytes were lysed in distilled water and proteins were precipitated by HClO_4_ (5%). After centrifugation, an aliquot of the supernatant (400 µL) was adjusted to pH 7.7 by the addition of saturated KHCO_3_ solution. All manipulations were performed at 4 °C to avoid ATP degradation. After dilution of the supernatant, the ATP concentration of the aliquots was determined utilizing the luciferin-luciferase assay kit (Roche Diagnostics, Mannheim, Germany) and a luminometer (Berthold Biolumat LB9500, Bad Wildbad, Germany) according to the manufacturer´s protocol.

### 3.6. Statistics

Data are expressed as arithmetic means ± SEM. As indicated in the figure legends, statistical analysis was made using ANOVA with Tukey’s test as the post-test and the *t*-test as appropriate. n denotes the number of different erythrocyte specimens studied. Since different erythrocyte specimens used in distinct experiments are differently susceptible to the triggers of eryptosis, the same erythrocyte specimens have been used for control and experimental conditions.

## 4. Conclusions

Shikonin stimulates Ca^2+^ entry and triggers ceramide formation, which, in turn, leads to shrinkage and phosphatidylserine translocation of erythrocytes. Accordingly, Shikonin triggers eryptosis, the suicidal erythrocyte death.
